# INTEGRATING AGE-FRIENDLY CARE INTO THE ONCOLOGY CONTINUUM

**DOI:** 10.1093/geroni/igad104.2614

**Published:** 2023-12-21

## Abstract

The number of older adults diagnosed with cancer is steadily rising, and measures for equitable, high-quality care in oncology are relatively undefined. The Association of Community Cancer Centers (ACCC), in collaboration with the Institute for Healthcare Improvement (IHI), led the first oncology-focused cohort guided by the 4Ms Framework for Age-Friendly Health Systems. Representatives from 22 cancer programs across the United States participated in a learning and action period to collaborate on building a program that provides optimal care for the older adult population. The Action Community consisted of a six-part didactic webinar series led by subject matter experts in geriatric oncology to apply the 4Ms model: What Matters, Mobility, Mentation, and Medication. ACCC asked sites to evaluate their program’s alignment with current standards using the ACCC Geriatric Gap Assessment tool and IHI’s 4Ms Care Description. Throughout the webinar series, participating sites in hospital and ambulatory settings identified a multidisciplinary team to improve workflows. The respondents answered questions regarding the frequency of assessments, documentation, and addressing inequities in their cancer program. Through a webinar poll, 90% of the participants from the multidisciplinary cancer team stated that medication was the most challenging “M” to implement and that further guidance from faculty experts was needed. More than half of the sites are progressing to earn level one (participant) or level two (committed to care excellence) recognition in the Age-Friendly Health Systems movement. ACCC has provided and will continue to provide health systems with resources for scale-up in their Age-Friendly journey.

